# Resident-Led Peer Support Groups in Emergency Medicine: A Pilot Framework for Peer Leader Training

**DOI:** 10.3390/bs15121744

**Published:** 2025-12-16

**Authors:** Kyra D. Reed, Alexandria P. Weston, Alexandra E. Serpe, Destiny D. Folk, Jacob M. Destrampe, Heather P. Kelker, Aloysius J. Humbert, Katie E. Pettit, Julie L. Welch

**Affiliations:** 1 Department of Emergency Medicine, Indiana University School of Medicine, Indianapolis, IN 46202, USA; dfolk@iu.edu (D.D.F.); jadest@iu.edu (J.M.D.); hpetrase@iu.edu (H.P.K.); ahumbert@iu.edu (A.J.H.); kburdick@iu.edu (K.E.P.); jlwelch@iu.edu (J.L.W.); 2 Department of Emergency Medicine, Indiana University Health Arnett Hospital, Lafayette, IN 47905, USA; aweston1@iuhealth.org; 3 Department of Emergency Medicine, Washington University in St. Louis School of Medicine, St. Louis, MO 63110, USA; serpe@wustl.edu

**Keywords:** peer support, peer leader, resident wellness, burnout, depression, emergency medicine

## Abstract

Peer support has demonstrated efficacy in alleviating symptoms of anxiety and depression, while fostering empathy and mitigating burnout among healthcare professionals. Given the considerable demands of residency training, there is a strong rationale for resident-led peer support interventions. However, structured programs to train residents for such leadership roles remain limited. Our objective was to implement a peer support leader training program for residents, evaluate its perceived effectiveness, and identify common themes discussed in sessions. Between June 2021 and June 2023, we performed a longitudinal, prospective cohort survey study of Emergency Medicine (EM) and EM/Pediatrics (EM/P) residents participating in a novel peer support leader training curriculum. Resident peer support leaders then facilitated biweekly support sessions, with post-session surveys assessing leader preparedness and themes discussed utilizing a novel Resident Stress Checklist (RSC). In total, 16 unique leaders were trained over two years and 52 biweekly peer support group sessions were held. In Year 1 (Y1), 6 resident leaders conducted an average of three sessions each, with 7 residents on average attending each session. In Year 2 (Y2), 4 leaders graduated and 2 leaders from Y1 continued in this role. An additional 10 resident leaders were trained. The 12 leaders in Y2 facilitated an average of 2 sessions each, with 5 residents on average attending each session. Of the completed post-session leader surveys (*n* = 39), 100% reported feeling prepared to lead the sessions based on their training. The RSC identified the most frequent stressor as work–life balance, most common symptom as frustration, and most common consequence of stress as emotional/psychological suffering. Resident-led peer support training was feasible and well-received, with all surveyed resident leaders reporting adequate preparation. The RSC revealed common session themes that guided future training topics and wellness curriculum interventions.

## 1. Introduction

A contemporary understanding of physician burnout encompasses three core dimensions: emotional exhaustion, depersonalization, and a diminished sense of accomplishment at work (Lacy & Chan, 2018). The Accreditation Council for Graduate Medical Education (ACGME) acknowledges physician well-being as a critical issue requiring urgent and sustained attention (ACGME, 2025). Current evidence indicates that burnout remains prevalent among medical residents, with estimates ranging from 35 to 52% (Lichstein et al., 2020; Low et al., 2019; Watling & Lingard, 2012). Rates are notably higher in high-intensity specialties such as emergency medicine (EM) (Hodkinson et al., 2022; Sakamoto et al., 2022). Among EM residents, burnout prevalence has been reported as high as 71%, and more specifically, having elevated levels of emotional exhaustion and depersonalization (Lacy & Chan, 2018). Moreover, burnout in EM residency has been associated with decreased job satisfaction, diminished professionalism, and compromised patient safety, including increased medication errors and reduced patient satisfaction (Hodkinson et al., 2022).

In the demanding context of EM training, residents frequently experience emotional distress and burnout. To address the well-documented prevalence of burnout among medical trainees (Kimo Takayesu et al., 2014), the implementation of peer support programs has emerged as a promising intervention (Abrams, 2017). These sessions have been integrated into residency programs to help trainees process the complex emotional and professional challenges inherent in their training (Rodrigues et al., 2018; Vongsachang & Jain, 2022). Prior studies have demonstrated that the involvement of peer or near-peer leaders increases participants’ willingness to engage in support groups (Mohamed et al., 2022; Jain et al., 2022; Vongsachang & Jain, 2022; Webber et al., 2021). This evidence suggests that peer leaders are particularly well-suited to facilitate these sessions as their presence promotes a sense of belonging and encourages open dialogue (Lee et al., 2023; Moore et al., 2020).

A qualitative study involving second-year residents identified peer learning as a key component in reducing distress among interns, ultimately recommending the development of a peer support skill-building curriculum to enable senior residents to more effectively support their colleagues (Moore et al., 2020). Additionally, survey data from peer support sessions indicates a strong preference for peer-led formats (Calder-Sprackman et al., 2018). Surveyed peer leaders also reported reciprocal benefits, including increased self-perceived empathy and efficacy following participation (Abrams, 2017). However, despite these findings, formal training programs designed for resident peer leaders remain scarce. Consequently, this lack of formalized training presents a barrier to the widespread implementation and consistency of peer support programs in residency. Without structured preparation, resident peer support leaders may struggle to navigate emotionally charged discussions, potentially limiting the effectiveness and psychological safety of these sessions.

The theoretical foundation for this study draws upon social support theory, which emphasizes the protective role of peer relationships in mitigating stress and fostering resilience in high-demand environments (Cohen & Wills, 1985). Within medical education, self-determination theory provides a complementary framework, highlighting how autonomy, competence, and relatedness contribute to intrinsic motivation and professional well-being (Ryan & Deci, 2000). These theories suggest that structured peer support can buffer emotional exhaustion and promote a sense of belonging among trainees. Additionally, the job demands-resources model (Demerouti et al., 2001) highlights the balance between occupational stressors and available supports. Interventions that enhance social resources, such as peer support programs, can counteract burnout. Collectively, these frameworks support the design of a formalized peer support leader curriculum aimed at enhancing well-being in residency training.

In addition to prior cross-sectional and intervention-based studies, recent literature highlights the value of longitudinal qualitative approaches in medical education. Such designs capture the evolving nature of trainee experiences, providing deeper insight into how wellness changes over time (Watling & Lingard, 2012). Longitudinal qualitative methods enable researchers to examine how contextual factors, such as workload, residency program culture, and peer relationships, influence well-being across multiple stages of training (Tavakol, 2019). This framework is particularly relevant for residency, where experiences and stressors evolve frequently. By incorporating longitudinal data collection and qualitative analysis, our study adds to this growing body of evidence and supports the development of sustained, resident-driven peer support initiatives.

Residency training presents unique challenges that require a tailored and integrated support system. In the absence of an established standardized peer support leader training model for EM residents, our objective was to develop, implement, and evaluate a formal curriculum to train resident peer leaders in a large EM residency at Indiana University School of Medicine, and identify common themes discussed. This initiative represents an innovative approach to promoting resident wellness and formal peer leader development within EM.

## 2. Methods

Study Design: From June 2021 to June 2023, we performed an IRB-approved, longitudinal, descriptive, prospective cohort survey study of 16 resident peer support leaders. We evaluated the effectiveness of their training, comfort leading sessions, and identified themes discussed during sessions.

Study Setting and Population: The peer support leaders included residents in the Emergency Medicine (EM) Residency (*n* = 63) and the Emergency Medicine/Pediatrics (EM/P) Residency (*n* = 10) at Indiana University School of Medicine for a total of 73 residents. The EM and EM/P residents rotate through three different academic, Level 1, high-volume training sites in Indianapolis, IN. These sites include a pediatric hospital (>50,000 visits per year), a county hospital (>100,000 visits per year), and a traditional academic hospital (>90,000 visits per year). On average, residents manage between 12 and 25 patients per 9 h shift, with PGY2s and above on the higher end. EM faculty oversee residents in the ED caring for patients, often managing patients individually as well.

Study Protocol: Volunteer peer support leaders were recruited via email sent to all eligible residents. Eligibility included any EM or EM/P residents post-graduate year (PGY) 2 or above and considered to be in good standing within the residency. Initially, 6 residents volunteered to be leaders for year 1 (Y1). Grant funding allowed for a small stipend to account for the residents’ time devoted to completing the training and leading sessions in Y1. An additional 10 peer support leaders volunteered the following year. In Y2, 4 leaders graduated and 2 continued in this role for a total of 12 leaders. Overall, there were 16 unique resident peer leaders trained over 2 years.

The resident peer leader training curriculum was based upon the successful EM faculty peer leader training program (Connors et al., 2023; Doehring et al., 2023) that utilized the American Medical Association guidelines for peer facilitators and resources from the National Alliance on Mental Illness (NAMI) (Connors et al., 2023; Jin & Chaudhari, 2023). This foundation was modified to reflect resident-specific scenarios and needs based on the available literature and iterative resident feedback from Y1 to Y2. Resident leader training included (A) asynchronous educational materials and (B) a one-hour virtual group training session (Table 1). The asynchronous portion consisted of several journal articles establishing the fundamentals of peer support programs and literature on resident burnout (de Haan et al., 2018; Hu, 2012; Shapiro, 2024). Additionally, webinars describing the benefits of peer support were provided (Shapiro, n.d.-a, n.d.-b). Peer leaders then met virtually with a trained faculty leader to discuss overall peer support session structure. The faculty leader was trained using the previous facilitator curriculum from the EM pilot studies (Connors et al., 2023; Doehring et al., 2023). During the virtual peer leader training session for residents, the faculty leader began with a discussion of the known benefits of peer support groups while reviewing the key points from the asynchronous assignments. Then, the structure and flow of peer support group sessions was explained, with additional training on each intentional component. The session included how to address safety planning for mental health emergencies and time to answer questions. All leaders were provided with a list of our site-specific mental health resources to share with residents at the end of the session.

After the training session, resident peer leaders created a voluntary sign-up sheet for the sessions on a shared document in a protected folder. This document contained dates, times, links (surveys, virtual meeting sites), number of participants that attended, and faculty backup call numbers in the event of questions or concerns. This document was kept up to date by the self-identified resident peer leader chairs. There was no requirement for residents leading a certain number of sessions. The one-hour resident-led peer support group sessions were held biweekly following scheduled resident didactics. Participation in the peer support group sessions by residents was voluntary and there were no minimum session requirements.

**Figure 1:**
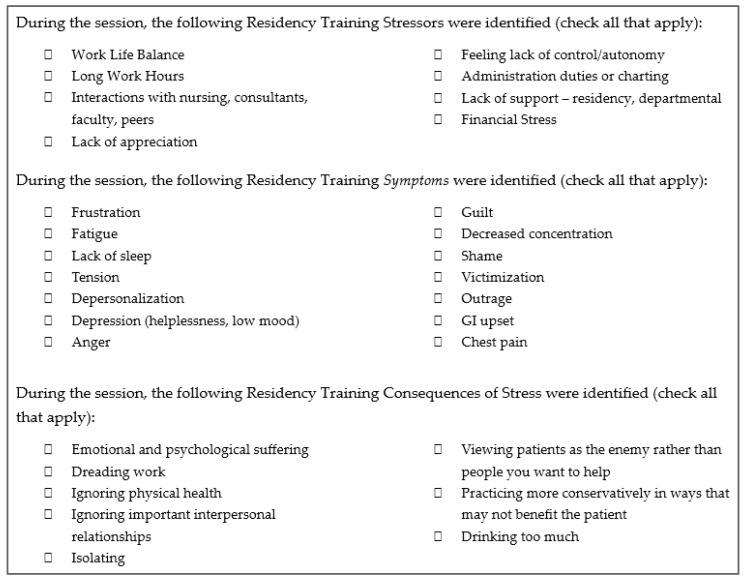
Modified Resident Stress Checklist.

Key Outcome Measures: Peer support leaders were invited to complete a post-session survey which asked: “Did you feel prepared or unprepared as the group leader?” The survey also included the Modified Resident Stress Checklist (Figure 1). This checklist was adapted from the Malpractice Stress Syndrome Scale (Kruczaj et al., 2023) and modified to include common residency training issues and needs from the available literature. The purpose of the checklist was to identify themes discussed during peer support sessions.

Additionally, peer support leaders were asked how many participants attended each session and to reflect on themes discussed and provide feedback for sessions. A focus group at the end of the year was held to reflect on the experience and provide feedback.

Data Collection and Analysis: After completing training, peer leaders signed up for scheduled sessions based on their availability. Although there were no specific assignments, peer leaders were encouraged to lead at least 2 sessions per year. The assigned leader communicated peer support group session date, time, and location to the residents through email, group text communication, and announcements during residency conferences. During the peer support group session, leaders first directed participants to a pre-session survey. Leaders then guided participants through the structured format, starting with check-ins, identifying themes, reframing challenges, and encouraging shared group wisdom. At the end of the session, leaders summarized what was discussed, reminded participants of mental health resources, and allowed time for post-session survey participation. The peer leaders also completed a post-session survey, documenting the number of attendees, themes discussed, preparedness, general reflections, and feedback for sessions.

Survey data were collected using an online survey created and administered via Qualtrics (Provo, UT). Descriptive quantitative and qualitative statistics were used to characterize peer support leader demographics, preparedness, and topics discussed during sessions derived from the Modified Resident Stress Checklist. Post-session feedback from peer leaders was reviewed by the faculty leader, who also serves as one of the residency assistant program directors, with iterative improvements made to both the residency program and leader training curriculum. The residency program improvements included decreasing or streamlining residents’ administrative tasks, adding content to the resident-nursing relations communication curriculum, serving breakfast at weekly didactics, creating a family planning elective for new parents, adding social events, and consolidating educational content to optimize time spent in the clinical learning environment. The resident peer leader training curriculum improvements included creating one QR code to all survey links for easier access, expanded discussion on helping a peer resident experiencing a mental health crisis during a peer support session, rules surrounding sharing about medical cases that are undergoing litigation, training on addressing microaggressions, and how to encourage participation with motivational interviewing techniques (e.g., open-ended questions, listening for understanding, reflection, validation, affirmation, summarization), (Table 1). Although a mental health crisis was never encountered in our study, this was a predominant source of hesitancy for resident peer leaders. After adding more information with step-by-step walk-through of how to address a mental health crisis, resident peer leaders reported feeling much more comfortable should this arise.

## 3. Results

Six residents volunteered to be peer support leaders for Y1 and 12 residents volunteered for Y2 for a total of 16 unique leaders (Table 2). A unique leader was defined as a single individual who had undergone leader training. Distinguishing unique leaders from total leaders accounted for addition, retention, and attrition of leaders from Y1 to Y2. Over the two-year period, 52 peer support sessions were held at least biweekly (virtual, in-person, or hybrid), with 232 total attendances (Y1 = 134, Y2 = 98). Y1 averaged three sessions per resident leader with seven participants per session and Y2 averaged two sessions per leader, with an average of five participants per session, respectively. No sessions were unfilled by a leader. The minimum and maximum number of sessions that one peer support leader held were 0 and 11, respectively. Demographics of the leaders were representative of this residency training program with 50–58%% female and 33% URM, with both percentages being slightly higher than the national average for EM residents reported in the literature during the timeframe of this study (Accreditation Council for Graduate Medical Education, 2023).

For Y1, of the 20 sessions held, 18 post-session leader surveys were completed (90% response rate), and of those leaders who answered the question regarding preparedness (*n* = 13), 100% felt prepared to lead sessions. For Y2, of the 26 sessions held, 24 post-session leader surveys were completed (92% response rate), and of those leaders answering the preparedness question (*n* = 24), 100% felt prepared to lead sessions. Thus, of those total leaders completing questions on preparedness for Y1 and Y2 (*n* = 37), 100% felt prepared by the training they had received (Table 3).

Leaders also reported the topics discussed during sessions using the Modified Resident Stress Checklist (Table 4). For total leader surveys submitted over the two-year period (*n* = 42), the most frequently identified residency stressors included: work–life balance (62%), long work hours (57%), and interactions with nursing/consultants/faculty/peers (55%). The symptoms of stress most reported included: frustration (76%), fatigue (71%), and lack of sleep (48%). The consequences of stress most frequently reported included: emotional/psychological suffering (52%), dreading work (50%), and ignoring physical health (40%).

Feedback from leaders on post-session surveys was positive, with leaders commenting that participants “are getting benefit from the sessions” with sessions ending on “positive notes,” and residents expressing it is helpful knowing “there is a light at the end of the tunnel” by hearing their peers at different levels of training to provide support and positive coping measures. Another leader reported that they “followed training videos and resources which lent itself to easy discussion facilitation.” Leaders provided recommendations on improving access to virtual links and surveys while also expressing interest in expanding leader training for addressing more specific scenarios like malpractice and suicidal ideation.

Additionally, from the peer support leader focus group session at the end of each year, one leader stated “this (being a peer support leader) was the most impactful thing I have done,” and another shared they were “impressed with how fast everyone took to it and how effective it was.” One leader stated, “I was surprised how much I got out of it even as the leader—cathartic.” Another leader shared they “felt overprepared for the sessions.”

## 4. Discussion

Our results demonstrate that we developed and implemented a successful peer support training program for our resident peer support leaders. Resident peer support leaders felt prepared for 100% of sessions and reported positive impact as a leader. Additionally, resident peer leader participation was a feasible time commitment, with an average of 2.9 sessions facilitated per resident leader over a year. The average number of five resident participants per session allowed our leaders to manage the session while maintaining fruitful conversations. Interestingly, 35% of peer support leaders identified as male in our study which is a large increase compared to a previous study where 100% of peer support leaders identified as female and more than 90% of peer support participants identified as female (Connors et al., 2023).

The results of our study regarding residents feeling prepared to lead peer support sessions after formal training builds upon the existing literature emphasizing the value of peer-led wellness initiatives. Moore et al. described a qualitative study of interns’ experiences of emotional distress and informal ad hoc peer support provided by senior residents. They proposed a model outlining how near peers can help mitigate distress, while recognizing the absence of training in their model to guide senior residents. They explicitly recommended the need for a formalized peer support skill-building curriculum during residency. Our study expands upon the Moore et al. model and operationalizes their recommendations by adapting, implementing, and evaluating a structured peer support leader training curriculum for EM residents. Augmenting Moore et al., our program provides a reproducible framework of (1) a standardized educational structure with synchronous and asynchronous components, including crisis safety planning and (2) a longitudinal evaluation of program outcomes using a post-session survey and novel Modified Resident Stress Checklist. In addition, unlike the ad hoc peer interaction described by Moore et al., our curriculum formalized training for peer leaders and demonstrated 100% leader-reported preparedness across 52 sessions spanning two years. This structured approach demonstrates feasibility and sustainability in a high acuity specialty and provides a model that may be adapted across other residency programs (Moore et al., 2020).

Reviewing the Leader Post-Session Survey, the session discussion topics reported on the Modified Resident Stress Checklist reveal the breadth of difficult conversations the leaders were required to address and discuss. Our results demonstrate that attendees felt comfortable discussing difficult topics with resident peer leaders which further supports existing research that peer leaders are helpful in facilitating peer support sessions as their presence fosters psychological safety for hard conversations (Lee et al., 2023; Moore et al., 2020; Vongsachang & Jain, 2022; Webber et al., 2021). These conversations are distinct opportunities for resident leaders to cultivate important communication and leadership skills, including the ability to validate feelings, reframe perspectives, share positive coping strategies, and navigate difficult conversations with their peers. All these vital skills will positively serve the residents long-term in not only their clinical duties with patients, colleagues, and nursing staff, but also in leadership roles throughout their careers. The peer support training combined with the in situ experience running sessions positioned resident leaders to be models of professionalism, confidentiality, and empathy by their peers, setting the tone for a culture in which trainees feel seen, validated, and understood.

To address the post-session feedback requesting further training on how to specifically navigate a peer exhibiting a mental health crisis, should such a situation arise, a detailed safety plan was added to the training program. Additionally, information was added on how to address malpractice issues in a supportive, yet legally approved way.

This study contributes to behavioral science by demonstrating the feasibility and effectiveness of a structured resident-led peer support training program for resident physicians. Methodologically, our approach combines longitudinal observation with post-session surveys to capture both skill acquisition and perceived impact over time, offering a model for evaluating peer-led interventions in clinical education settings. By systematically measuring preparedness, participation, and session outcomes, this study provides a framework for assessing peer support programs across specialties, addressing a gap in empirical evaluation of peer-led behavioral interventions in medical training.

The findings have several actionable implications for residency programs. Formal training combined with in-person experience equips resident leaders to facilitate psychologically safe sessions and navigate difficult conversations, enhancing both communication and leadership skills. Programs can adopt our structured session schedule, aiming for small group sessions to balance feasibility with meaningful discussion. Targeted safety and legal guidance ensure that resident leaders are prepared to handle mental health crises appropriately. Collectively, these results offer replicable strategies for residency programs to implement or enhance peer support initiatives, further supporting trainee well-being and professional development.

## 5. Limitations

One limitation of this study is the number of peer support leaders. Although 16 leaders volunteered to lead these sessions, more leaders undergoing this leader training would improve our understanding of the effectiveness and needs of the training curriculum. Individual peer support leaders likely require different training material and methods to feel prepared and successful. While 100% of the leaders in this study felt prepared, an increased number of leaders could give a better understanding of additional individual needs. Another limitation is that this training was performed at a single institution with residents from two training programs. Residency training challenges and curricula are unique to each training program, so the approach we have described in this study may not be broadly applicable. Additionally, survey studies inherently have response and selection bias that must be considered when interpreting results. Our survey responses and completion could have been influenced by stigma regarding mental health. Finally, it would be helpful to have had pre- and post-data from the training session to test understanding and retention of concepts.

## 6. Conclusions

We successfully trained 16 peer support leaders with our novel training curriculum. 100% of leaders felt prepared to navigate peer support contributing to the current literature on the topic. Leaders also reported individual benefits to leading peer support sessions including improvement in their ability to navigate difficult conversations with patients as well as peers. Further research should expand the training curriculum to additional residency programs and institutions to further assess and refine the overall content and improve generalizability. In addition, further research could explore the impact of peer support leadership training and experience on communication skills with patients, career satisfaction, and career burnout.

## Figures and Tables

**Table 1:** Peer Support Group Training Curriculum.

Peer Support Educational Method	Educational Materials and Content
Asynchronous Education	Review articles (Grech, 2021; Hu, 2012; Shapiro, 2024) Webinars (Shapiro, n.d.-a, n.d.-b)
B. Synchronous Group Training Session (virtual), 1 h	Welcome Study Information, Consents Review Asynchronous Training Materials Nuts and Bolts of Peer Support Group Structure a. Opening: Introductions, Sharing recent highs and lows b. Empathetic listening, Validation c. Reflecting on themes shared d. Reframing strategies e. Encouraging group wisdom f. Positive coping strategies g. Mental Health Emergencies and Protocols h. Resource list i. Positive Closing: What the participant took away or something they are looking forward to Survey dissemination logistics Session frequency and leader sign-ups Questions Year 2 (Y2) Educational Content Additions: Important Scenarios/Topics a. Motivational Interviewing Techniques b. Malpractice/Lawsuits: Rules c. Additional Mental Health Crisis Education d. Diversity, Equity, and Belonging Overview of Year 1 (Y1) Results and Feedback a. Survey QR Codes Y1 Leader Panel a. General reflections b. What I wish I had known c. Tips on logistics of running sessions

**Table 2:** Peer Support Group Resident Peer Leader Demographics and Metrics.

	Year 1				Year 2				Year 1 + Year 2			
	Leaders	Sessions Led	Sessions Led Average	Attrition	New Trained Leaders	Leaders	Sessions Led	Sessions Led Average	Total Leaders	Unique Leaders	Sessions Led	Sessions Led Average
	*n (%)*	*n (%)*	*n (min−max)*	*n (%)*	*n*	*n (%)*	*n (%)*	*n (min–max)*	*n*	*n*	*n (%)*	*n (min–max)*
Total						—						
	6	20	3.3	4	10	12	26	2.2	*	16	46	2.9 (0–13)
PGY												
1	0 (0%)	0 (0%)	0 (0–0)	0 (0%)	0	0 (0%)	0 (0%)	0 (0–0)	0	*	0 (0%)	0 (0–0)
2	1 (17%)	4 (20%)	4 (4–4)	0 (0%)	7	7 (58%)	19 (73%)	2.7 (0–13)	11		23 (50%)	2.1 (0–13)
3–5	5 (83%)	16 (80%)	3.2 (2–4)	4 (67%)	3	5 (42%)	7 (27%)	1.0 (0–2)	10		23 (50%)	2.3 (0–4)
Gender												
Male	3 (50%)	10 (50%)	3.3 (2–4)	2 (50%)	4	5 (42%)	6 (23%)	1.2 (0–2)	8	7	16 (35%)	2 (0–4)
Female	3 (50%)	10 (50%)	3.3 (3–4)	2 (50%)	6	7 (58%)	20 (77%)	2.9 (0–13)	10	9	30 (65%)	3 (0–13)
URM												
Yes	2 (33%)	7 (35%)	(3–4)	2 (50%)	4	4 (33%)	4 (15%)	1.0 (0–2)	6	6	11 (28%)	1.8 (0–4)
No	4 (67%)	13 (65%)	(2–4)	2 (50%)	6	8 (67%)	22 (85%)	2.8 (0–13)	12	10	35 (76%)	2.9 (0–13)

PGY = post-graduate year. URM = underrepresented in medicine. * = Unable to calculate due to duplicates of unique leaders and retention of leaders through multiple levels of training.

**Table 3:** Post-Session Resident Peer Leader Survey: Preparedness.

	Year 1 (Y1) (*n* = 13 Responses)	Year 2 (Y2) (*n* = 24 Responses)
Peer Leader Post-Session Survey Question	Did you feel prepared or unprepared as the group leader?	I felt prepared to lead this peer support session.
Prepared/Yes	13 (100%)	24 (100%)
Unprepared/No	0 (0%)	0 (0%)

**Table 4:** Post-Session Leader Survey: Modified Resident Stress Checklist.

Residency Training Stressor: “During the Session, the Following Residency Training Stressors Were Identified (Check All That Apply):”			
Topic	Year 1 (Y1) (*n* = 18) *n* (%)	Year 2 (Y2) (*n* = 24) *n* (%)	Total Y1 + Y2 (*n* = 42) *n* (%)
Work Life Balance	11 (61%)	15 (63%)	26 (62%)
Long Work Hours	6 (33%)	18 (75%)	24 (57%)
Interactions with nursing, consultants, faculty, peers	11 (61%)	12 (50%)	23 (55%)
Lack of appreciation	5 (28%)	5 (21%)	10 (24%)
Feeling lack of control/autonomy	4 (22%)	5 (21%)	9 (21%)
Administration duties or charting	4 (22%)	5 (21%)	9 (21%)
Lack of support—residency, departmental	5 (28%)	0 (0%)	5 (12%)
Financial Stress	2 (11%)	1 (4%)	3 (7%)
Residency Symptoms of Stress: “During the session, the following Residency Training Symptoms of Stress were identified (check all that apply):”			
Frustration	14 (78%)	18 (75%)	32 (76%)
Fatigue	12 (67%)	18 (75%)	30 (71%)
Lack of sleep	7 (39%)	13 (54%)	20 (48%)
Tension	7 (39%)	8 (33%)	15 (36%)
Depersonalization	6 (33%)	6 (25%)	12 (29%)
Depression (helplessness, low mood)	5 (28%)	5 (21%)	10 (24%)
Anger	6 (33%)	3 (13%)	9 (21%)
Guilt	5 (28%)	2 (8%)	7 (17%)
Decreased concentration	3 (17%)	2 (8%)	5 (12%)
Shame	3 (17%)	1 (4%)	4 (10%)
Victimization	3 (17%)	1 (4%)	4 (10%)
Outrage	0 (0%)	0 (0%)	0 (0%)
GI upset	0 (0%)	0 (0%)	0 (0%)
Chest pain	0 (0%)	0 (0%)	0 (0%)
Residency Consequences of Stress: “During the session, the following Residency Training Consequences of Stress were identified (check all that apply):”			
Emotional and psychological suffering	8 (44%)	14 (58%)	22 (52%)
Dreading work	11 (61%)	10 (42%)	21 (50%)
Ignoring physical health	9 (50%)	8 (33%)	17 (40%)
Ignoring important interpersonal relationships	5 (28%)	6 (25%)	11 (26%)
Isolating	4 (22%)	4 (17%)	8 (19%)
Viewing patients as the enemy rather than people you want to help	3 (17%)	2 (8%)	5 (12%)
Practicing more conservatively in ways that may not benefit the patient	3 (17%)	1 (4%)	4 (10%)
Drinking too much (alcohol)	0 (0%)	0 (0%)	0 (0%)

## Data Availability

The datasets presented in this article are not readily available due to participant privacy.

## Abbreviations

The following abbreviations are used in this manuscript:

EM	Emergency Medicine
EM/P	EM/Pediatrics
RSC	Resident Stress Checklist
Y1	Year One
Y2	Year Two
ACGME	Accreditation Council for Graduate Medical Education
PGY	Post-Graduate Year
NAMI	National Alliance on Mental Illness
